# Effects of supplementing with *Nigella sativa* meal and selenium nano-particles on growth performance, immunity, microbial count, oxidative stability, and intestinal integrity-related gene expression in heat-stressed growing rabbits

**DOI:** 10.1007/s11259-025-11026-x

**Published:** 2026-01-22

**Authors:** Ahmed M. Elbaz, Bahaa Farrag, B. F. Farag, Abdel‑Moneim Eid Abdel‑Moneim

**Affiliations:** 1 Animal and Poultry Nutrition Department, Desert Research Center, Mataria, Cairo, Egypt; 2 Animal and Poultry Physiology Department, Desert Research Center, Mataria, Cairo Egypt; 3 Animal Production Department, Faculty of Agriculture, Assiut Branch, Al-Azhar University, Cairo , Egypt; 4 Biological Applications Department, Nuclear Research Center, Egyptian Atomic Energy Authority, Cairo, Egypt

**Keywords:** Gene expression, Heat stress, *Nigella sativa* meal, Rabbits' performance, Selenium

## Abstract

Heat stress severely impacts the performance of growing rabbits, compromising their survival and hampering the sustainability of rabbit farming. Therefore, this study investigated the effects of nano-selenium, *Nigella sativa* meal, and their mixture supplementation on immune responses, antioxidant activity, gut health, and growth performance in growing rabbits. One hundred male New Zealand White rabbits were equally divided into four groups, each with five replicates at 35 days of age (5 rabbits/replicate). The control group was fed on a basal diet (CON) while the other groups were fed a basal diet supplemented with *Nigella sativa* meal (NSM, 40 g/kg), Nano-selenium (Nano-Se, 0.1 mg/kg), and their mixture (Se-NSM), respectively. Results showed that exposure of growing rabbits to heat stress resulted in a significant deterioration in growth performance and immune-oxidative status. Additionally, feeding rabbits with Se-NSM enhanced body weight gain, carcass weight, and feed conversion ratio. Se-NSM supplementation increased the digestibility of dry matter, crude fiber, and crude protein, and increased the activity of lipase and trypsin enzymes. Superoxide dismutase (SOD), total antioxidant capacity (TAC) activity, and triiodothyronine (T3), IgA, and IgG levels increased, while malondialdehyde (MDA) decreased in rabbits receiving Se-NSM supplementation. Adding Se-NSM decreased triglycerides, cholesterol, and LDL levels, while increasing glucose, total protein, and AST levels in rabbits. Supplementing Se-NSM enhanced gut health via reducing pathogenic microbes counts and mucin-2 (MUC-2) and cationic amino acid transporter-1 (CAT-1) gene expression. These findings indicate that combining Nano-selenium-*Nigella sativa* meal enhanced the rabbits’ growth performance, immune response, and gut integrity during heat stress.

## Introduction

Most countries around the world have banned the use of antibiotics due to their potential risks to both the animal and the consumer (Abdel-Moneim et al. 2020). However, many rabbit breeders are still forced to use them because of the digestive and intestinal problems rabbits suffer from during the weaning period, which leads to poor performance and health, high mortality rates, and significant economic losses (Elbaz et al. 2023b). Furthermore, rabbits suffer from the risk of heat stress during the summer season due to their inability to adapt to high temperatures, resulting in digestive and physiological disturbances and leading to poor performance (Abdel-Moneim et al. 2021; Attia et al. 2017). The most important symptoms of heat stress include microbial imbalance, oxidative stress, immunodeficiency, and increased intestinal inflammation (Abdel-Moneim et al. 2022; El-Baz and Khidr 2024). Therefore, there is an urgent need to select feed additives to support the health and safety of rabbits by developing the immune system and strengthening the intestinal barrier. This is achieved by modifying the microbial content of the host’s gut to eliminate harmful microbes, thereby enhancing intestinal health and immune response (Ibrahim et al. 2020; Fathi et al. 2023), in addition to modifying immunity and reducing oxidative stress (Elbaz et al. 2022).

During the production of essential oils from medicinal and aromatic plants, a substantial quantity of by-products is generated as waste (Saha et al. 2018). These by-products are scientifically important because they often contain valuable bioactive compounds, such as phenolic, flavonoids, and residual essential oils, which can be utilized in animal nutrition as natural antioxidants, antimicrobials (Attia and Al-Harthi 2015; Wang et al. 2018), or feed additives, thereby reducing environmental waste and enhancing resource sustainability. Black seed meal, a major by-product of essential oil extraction, accounts for approximately 70–75% of the total weight of black seed fruit (Fathi et al. 2023) and can be utilized as a supplement or as a substitute for conventional feed ingredients (Elbaz et al. 2023c). *Nigella sativa* meal is a perfect source of important nutrients, including protein, fat, and essential amino acids (Elbaz 2023; Fathi et al. 2023; Elbaz et al. 2025d), as well as being a good source of biologically active compounds, prebiotics, and polysaccharides. *Nigella sativa* contains several biologically active components, including thymoquinone, thymol, dithymoquinone, p-cymene, and carvacrol (Dalli et al. 2021), which contribute to its powerful antioxidant, anti-inflammatory, antimicrobial, and immunomodulatory properties. Additionally, numerous extensive studies have been conducted on *Nigella sativa* (black seed) to explore its pharmacological effects, including antimicrobial, antioxidant, anti-inflammatory, immunomodulatory, bronchodilator, gastro-protective, and other properties (Fathi et al. 2023), which is due to the biologically active ingredients. Several previous reports have demonstrated the positive effects of *Nigella sativa* supplementation on the performance, immunity, and overall health of rabbits and broiler chickens (Attia and Al-Harthi 2015; Demirci et al. 2019).

Selenium is one of the most important nutrients essential for the normal functioning of the immune system, maintaining organ function, intestinal health, and bird performance (Ibrahim et al. 2020; Kassim et al. 2022; Elbaz et al. 2025a). Numerous studies have shown that selenium deficiency leads to major digestive disorders, polycystic ovary syndrome, pancreatic fibrosis, liver damage, and impaired thyroid metabolism (Kieliszek and Błażejak 2016), leading to poor growth performance. Furthermore, adding selenium to rabbit feed contributes to maintaining good health and productive performance (Sheiha et al. 2020). Furthermore, nanotechnology provides effective solutions for improving health and nutrient delivery, protecting the health and performance of poultry production (Ibrahim et al. 2020; Kassim et al. 2022). Interest in nanoparticles is due to their unique physical and chemical properties, which include high bioavailability, high stability, large surface area, high adsorption capacity, hydrophobicity, small size, and low toxicity (Ibrahim et al. 2020; Abdel-Moneim et al. 2022). In addition, previous reports indicated that adding selenium nanoparticles (Nano-Se) as an additional source of trace minerals in feeds enhanced the immune response, intestinal health, and performance of chickens (Sheiha et al. 2020; Ayyat et al. 2018).

In the current study, *Nigella sativa* meal (black seed) and nano-selenium were chosen as feed additives for growing rabbits. These potential additives can effectively mitigate the negative effects of stress and weaning complications, based on the documented advantages of each in numerous previous reports. Several studies have evaluated the effect of adding nano-selenium supplements or *Nigella sativa* powder on the performance and health of growing rabbits, while there are no studies examining the effect of a dietary combination of nano-selenium supplements or black seed powder on rabbit performance under hot environmental conditions. Therefore, this study examined the impact of supplementing *Nigella sativa* meal, Nano-selenium, and their mixture in growing rabbit feed on performance, antioxidant status, immunity, mucin-2 and cationic amino acid transporter-1 gene expression, and intestinal microbial counts of rabbits raised in hot environmental conditions.

## Materials and methods

### Experimental design and diets

One hundred male New Zealand White rabbits, aged 35 days, were randomly assigned to four experimental groups (five replicates, 5 rabbits/replicate), with a mean starting weight of 731.6 ± 13.4 g. The groups were as follows: the control group (CON) was fed a basal diet; the second, third, and fourth groups were fed a basal diet containing *Nigella sativa* meal (NSM, 40 g/kg), Nano-selenium (Nano-Se, 0.1 mg/kg), or their mixture (Se-NSM), respectively. Experimental rabbits were housed under uniform management conditions in a well-ventilated room during the summer. To meet the nutritional needs of growing experimental rabbits, basal diets were formulated based on the recommendations of the National Research Council (NRC, 1977), as presented in Table 1. Pelleted feed and clean fresh water were provided *ad libitum* throughout the experimental period. Temperature and humidity were recorded daily; the average temperature was 30.9 °C, and the humidity was 52% during the experimental period. The experiment was conducted in the summer of 2024. *Nigella sativa* meal was purchased from ALHAWAG Company, Giza, Egypt, and analyzed at the Central Laboratory of the Desert Research Center, as described by Elbaz et al. (2025d). Nano-selenium was obtained from the National Research Center, Dokki, Cairo, Egypt. Chemical analysis of *Nigella sativa* meal revealed that it consisted of 90.8% dry matter (#934.01), 26.3% crude protein (#984.13), 7.1% crude fiber (#978.10), and 2.4% crude fat (#920.39), according to AOAC (2002).

**Table 1:** Ingredients and chemical composition of experimental diets

Ingredients	%
Yellow corn	15.3
Soybean meal (44% CP)	17.2
Alfalfa dehydrated	39.5
Sunflower meal	5.00
Wheat bran	10.0
Barley	9.00
Limestone	0.50
Premix (Vit-Min)*	0.50
Di-Calcium phosphate	0.70
Salt	0.30
Molasses	2.00
Nutritive value (%)	
Organic matter	91.82
Crude protein	18.36
Crude fiber	13.74
Calcium	1.16
Phosphorus	0.54
Metabolizable energy	2500

*Each 1 kg of vitamin-mineral premix contained: 5.46 gm of phylloquinone, 131.30 gm of DL-3-tocopheryl acetate, 5.64 gm of thiamine, 14.56 gm of riboflavin, 7.35 gm of pyridoxine, 27.30 gm of Ca-D-pantothenate, 3.64 gm of folic acid, 109.20 gm of niacin, 29.12 mg of cobalamin, 120 gm of manganese, 3.00 gm of selenium, 237 mg of D-biotin, 900 mg of zinc, 160 mg of copper, 12.50 gm of iodine, and 400 mg of ferrous

### Performance indices

At the end of the experiment (77 days of age), the live body weight (LBW) and feed intake (FI) were recorded weekly. Body weight gain (BWG) and feed conversion ratio (FCR, FI/BWG) were calculated at the end of the trial period. To evaluate carcass characteristics at 77 days of age, one growing rabbit was randomly selected from each experimental replicate (5 rabbits/group), weighed individually, and then slaughtered. Carcass characteristics include the carcass, heart, liver, lungs, kidneys, and viscera weighed, and their relative weights to the live body weight were calculated. Additionally, samples of small intestine contents were taken during slaughter to estimate the activities of digestive enzymes (amylase, trypsin, and cellulase). The samples were immediately frozen in liquid nitrogen and stored at − 20 °C until analysis. Commercial kits (Nanjing Jiancheng Bioengineering Institute, China) were used to estimate the activity of digestive enzymes as described by Elbaz et al. (2023a). After the experimental period, one animal/replicate (5 rabbits/group) was separated into metabolic cages and starved to begin the digestion experiment for 12 h. Feces were collected twice daily for 4 days, dried (65 °C for 48 h), ground, and stored until the target chemical analyses were performed. Feed and fecal samples were analyzed for dry matter (DM), crude fiber (CF), crude protein (CP), ether extract (EE), and nitrogen-free extract (NFE) contents, according to the methods of AOAC (2000). Although no chemical analysis of the active compounds has been performed, previous studies have reported that the phytochemical composition of *Nigella sativa* contains biologically active compounds, including thymoquinone, thymohydroquinone, carvacrol, and para-cymene (Dalli et al. 2021; Ouattar et al. 2022).

### Blood biochemistry

During slaughtering (at 77 weeks), blood samples were collected in anticoagulant-free tubes and centrifuged at 3500 × g for 10 min, and serum was collected and stored at −20 °C until analyzed. Glucose, triglycerides, low-density lipoprotein (LDL), cholesterol, high-density lipoprotein (HDL), alanine aminotransferase (ALT), aspartate aminotransferase (AST), total protein, and albumin levels were measured calorimetrically using an auto-analyzer system by using commercially available kits and following the manufacturer’s instructions (Spinreact Co., Girona, Spain). The levels of IgM, IgG, and IgA in blood were determined by enzyme-linked immunosorbent assay (ELISA). Triiodothyronine (T3) was assayed using radioimmunoassay (RIA) kits, as explained by Elbaz et al. (2023b). Serum superoxide dismutase (SOD), total antioxidant capacity (TAC), and malondialdehyde (MDA) levels were specified according to the instructions of the commercial kits (Shanghai Zhucai Biotechnology Co., China).

### Microbial content

At slaughter, 10 g of the cecum of five animals/group was collected, and the samples were stored at −20 °C after being placed in sterile bags until the required analysis. Dilutions of the cecal samples were made as necessary and cultivated on agar appropriate for each microbe at the required temperature and under aerobic or anaerobic conditions. *Clostridium perfringens*, *Lactobacillus*, and *Escherichia coli* were counted after incubation on egg yolk emulsion (50%), MRS agar, and MacConkey agar, respectively, as explained by Elbaz et al. (2025a). The enumeration of microbiota was evaluated as log 10 colony-forming units per gram of cecal digesta.

### Gene expression

At the end of the experimental period, 20 growing rabbits (5 rabbits per group) were sacrificed for cecal tissue sampling to estimate the effect of experimental supplementation on the mucin-2 (MUC-2) and cationic amino acid transporter-1 (CAT-1) gene expression. Total RNA suspension was extracted from the cecal membranes and then homogenized, visualized, and quantified using a Nanodrop spectrophotometer (BMG Lab Tec. GmbH, Germany); all procedures were performed according to the manufacturer’s instructions. After the amplification of cDNA, the specificity of the amplification was assessed by performing a dissociation curve of the real-time polymerase chain reaction (7500 Fast Real-time PCR) products. The forward and reverse primers for MUC-2: F: TATACCGCAAGCAGCCAGGT and R: GCAAGCAGGACACAGACCAG (Accession number L41544.1); and CAT-1 F: CCAGTCTATTAGGTTCCATGTTCC and R: CGATTATTGGCGTTTTGGTC (XM_002721425.3), respectively. The 2 − ΔΔCT method was used to determine the relative expression levels of the MUC2 and CAT-1 gene (Livak and Schmittgen 2001).

### Statistical analysis

For all experimental data analysis, a one-way analysis of variance (ANOVA) was used using the statistical program SPSS (version 21; SPSS Inc., Chicago, IL, USA). Tukey’s test was used to determine the significance of the mean differences, and the results are presented as mean ± standard deviation (± SD) to compare the differences among experimental groups. All differences were considered statistically significant when *P* < 0.05.

## Results

### Performance indicators

The effects of dietary *Nigella sativa* meal and nano-selenium supplementation on growth performance and carcass traits of growing rabbits under hot environmental conditions are presented in Tables 2 and 3. The obtained results showed that feeding rabbits diets with NSM, Nano-Se, or Se-NSM had a significant effect on growth performance compared to the control group. Live body weight and body weight gain increased in rabbits fed supplements NSM, Nano-Se, and Se-NSM (*P* < 0.05) compared to the control group, while the Se-NSM group showed the best live body weight and body weight gain (*P* < 0.05). On the other hand, NSM, Nano-Se, and Se-NSM supplementation tended to decrease (*P* < 0.05) the feed conversion ratio compared to the rabbits group fed the control diet; however, the feed conversion ratio was lower in rabbits receiving Se-NSM. Furthermore, the experimental supplements did not affect daily feed intake (*P* < 0.05).

**Table 2:** Effect of *Nigella sativa* meal and Nano-selenium-supplementation on growth performance in growing rabbits under hot environmental conditions

Parameters	CON	NSM	Nano-Se	Se-NSM	*P* value
Initial body weight (g, 35 d)	731 ± 1.97	736 ± 1.86	729 ± 2.91	733 ± 1.72	0.317
Live body weight (g, 77 d)	1785 ± 7.08^c^	1876 ± 6.12^b^	1854 ± 8.04^b^	1963 ± 9.23^a^	< 0.001
Body weight gain (g/d)	24.13 ± 0.29^c^	26.04 ± 0.33^b^	25.92 ± 0.41^b^	28.31 ± 0.27^a^	< 0.001
Daily feed intake (g/d)	83.8 ± 2.16	84.5 ± 1.54	84.2 ± 2.31	84.7 ± 3.01	0.225
Feed conversion ratio (g/g)	3.49 ± 0.02^a^	3.26 ± 0.05^b^	3.25 ± 0.01^b^	3.02 ± 0.04^c^	< 0.001

Growing rabbits were given a basal diet (CON, without feed additive), or supplemented with *Nigella sativa* meal (NSM, 40 g/kg), supplemented with Nano-selenium (Nano-Se, 0.1 mg/kg), supplemented with *Nigella sativa* meal and Nano-selenium (Se-NSM); Means with different superscripts within the same row differ significantly (a–c, *p* < 0.05); All data are expressed as the mean ± SD

**Table 3:** Effect of *Nigella sativa* meal and Nano-selenium-supplementation on carcass traits (%) in growing rabbits under hot environmental conditions

Parameters	CON	NSM	Nano-Se	Se-NSM	*P* value
Live body weight (g)	1756 ± 6.32^c^	1839 ± 7.28^b^	1841 ± 8.36^b^	1958 ± 9.11^a^	< 0.001
Carcass	55.8 ± 5.41^c^	61.3 ± 6.14^ab^	58.4 ± 7.02^b^	63.1 ± 6.29^a^	0.001
Heart	0.31 ± 0.12	0.30 ± 0.23	0.29 ± 0.12	0.31 ± 0.25	0.522
Liver	3.19 ± 0.54	3.43 ± 0.61	3.32 ± 0.70	3.28 ± 0.44	0.317
Kidneys	0.58 ± 0.09	0.56 ± 0.11	0.57 ± 0.06	0.53 ± 0.08	0.230
Lungs	0.61 ± 0.08	0.60 ± 0.05	0.63 ± 0.09	0.61 ± 0.06	0.431
Edible giblet	4.17 ± 0.14	4.09 ± 0.20	4.13 ± 0.18	4.07 ± 0.34	0.185

Growing rabbits were given a basal diet (CON, without feed additive), or supplemented with *Nigella sativa* meal (NSM, 40 g/kg), supplemented with Nano-selenium (Nano-Se, 0.1 mg/kg), supplemented with *Nigella sativa* meal and Nano-selenium (Se-NSM); Means with different superscripts (a–d) within the same row differ significantly (a–d, *p* < 0.05); All data are expressed as the mean ± SD

Additionally, the characteristics of the carcass were not influenced by the experimental treatments, except for the carcass weight. Carcass weight increased (Table 3, *P* < 0.05) in rabbits fed diets containing NSM, Nano-Se, and Se-NSM compared to rabbits fed diets without supplements; however, the carcass weight was higher in rabbits receiving Se-NSM.

### Digestive performance

The effects of dietary *Nigella sativa* meal and nano-selenium supplementation on nutrient digestibility and digestive enzyme activity of growing rabbits under hot environmental conditions are presented in Table 4. The results showed significant changes in the activity of digestive enzymes, increased lipase and trypsin activity (*P* < 0.05) in the blood of rabbits fed a diet containing NSM and Se-NSM compared to rabbits fed a diet containing Nano-Se and the control diet, while amylase activity was not be affected by the experimental treatments (*P* < 0.05).

**Table 4:** Effect of *Nigella sativa* meal and Nano-selenium-supplementation on nutrient digestion and digestive enzymes activity in growing rabbits under hot environmental conditions

Parameters	CON	NSM	Nano-Se	Se-NSM	*P* value
Enzymes activity					
Lipase (U/g)	62.7 ± 1.33^b^	66.3 ± 0.95^a^	63.4 ± 2.09^b^	67.4 ± 1.52^a^	0.001
Trypsin (KU/mg)	1.93 ± 0.04^b^	2.24 ± 0.07^a^	2.01 ± 0.02^b^	2.31 ± 0.06^a^	0.001
Amylase (U/g)	2.68 ± 0.05	2.71 ± 0.03	2.66 ± 0.08	2.69 ± 0.05	0.344
Nutrient digestion					
Dry matter	59.8 ± 1.34^b^	63.7 ± 0.99^a^	60.9 ± 1.16^b^	64.1 ± 0.82^a^	0.001
Crude fiber	41.3 ± 0.83^c^	44.8 ± 1.45^a^	42.9 ± 1.01^b^	45.6 ± 0.73^a^	< 0.001
Crude protein	64.5 ± 1.01^c^	68.2 ± 1.21^b^	66.5 ± 2.17^bc^	72.5 ± 1.34^a^	< 0.001
Ether extract	78.3 ± 2.18	79.1 ± 2.67	78.5 ± 1.85	79.4 ± 1.64	0.176
NFE	54.9 ± 1.41	55.4 ± 1.94	55.2 ± 2.30	55.7 ± 1.72	0.402

Growing rabbits were given a basal diet (CON, without feed additive), or supplemented with *Nigella sativa* meal (NSM, 40 g/kg), supplemented with Nano-selenium (Nano-Se, 0.1 mg/kg), supplemented with *Nigella sativa* meal and Nano-selenium (Se-NSM); Means with different superscripts within the same row differ significantly (a–c, *p* < 0.05); All data are expressed as the mean ± SD

Nutrient digestibility was significantly influenced by the experimental supplements, with dry matter digestibility increasing (Table 4, *P* < 0.05) in rabbits fed a diet containing NSM and Se-NSM compared to those fed a diet with Nano-Se and the control diet. Crude protein digestibility increased (*P* < 0.05) in rabbits fed a diet containing NSM, Nano-Se, and Se-NSM compared to rabbits fed the control diet; however, the crude protein digestibility was higher in rabbits receiving NSM and Se-NSM. Crude fiber digestibility increased (*P* < 0.05) in rabbits fed a diet containing NSM, Nano-Se, and Se-NSM compared to rabbits fed the control diet. The experimental supplements did not affect the Ether extract and NFE digestibility (*P* < 0.05).

### Blood biochemical constituents

**Fig. 1:**
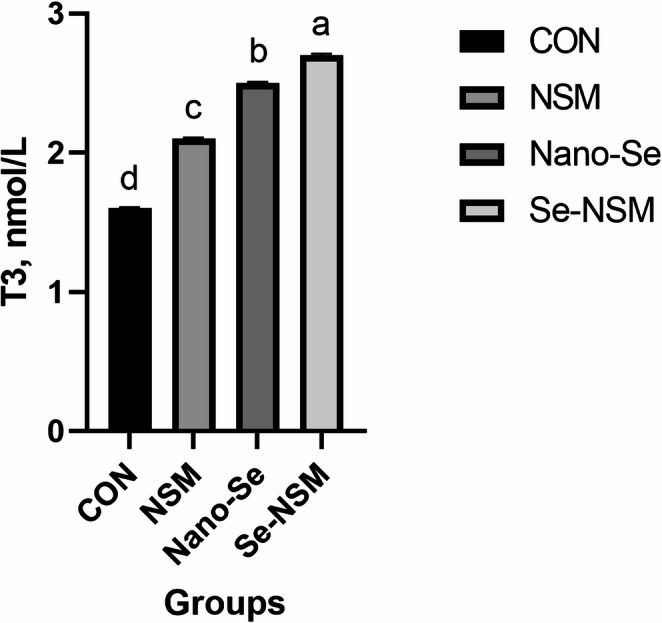
Effect of adding *Nigella sativa* meal and Nano-selenium on triiodothyronine (T3) levels in growing rabbits under hot environmental conditions. Growing rabbits were given a basal diet (CON, without feed additive), or supplemented with *Nigella sativa* meal (NSM, 40 g/kg), supplemented with Nano-selenium (Nano-Se, 0.1 mg/kg), supplemented with *Nigella sativa* meal and Nano-selenium (Se-NSM); Means with different superscripts within the same row differ significantly (a–d, *p* < 0.05); All data are expressed as the mean ± SD

**Fig. 2:**
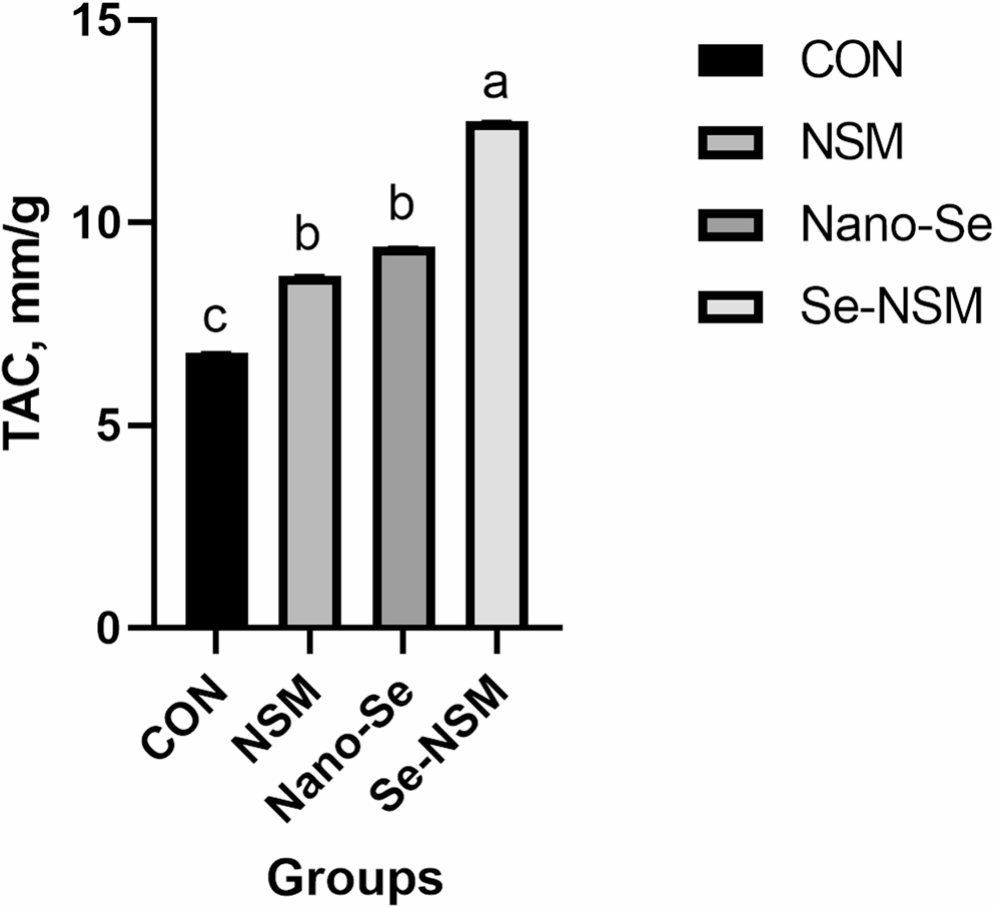
Effect of adding *Nigella sativa* meal and Nano-selenium on total antioxidant capacity (TAC) levels in growing rabbits under hot environmental conditions. Growing rabbits were given a basal diet (CON, without feed additive), or supplemented with *Nigella sativa* meal (NSM, 40 g/kg), supplemented with Nano-selenium (Nano-Se, 0.1 mg/kg), supplemented with *Nigella sativa* meal and Nano-selenium (Se-NSM); Means with different superscripts within the same row differ significantly (a–d, *p* < 0.05); All data are expressed as the mean ± SD

**Fig. 3:**
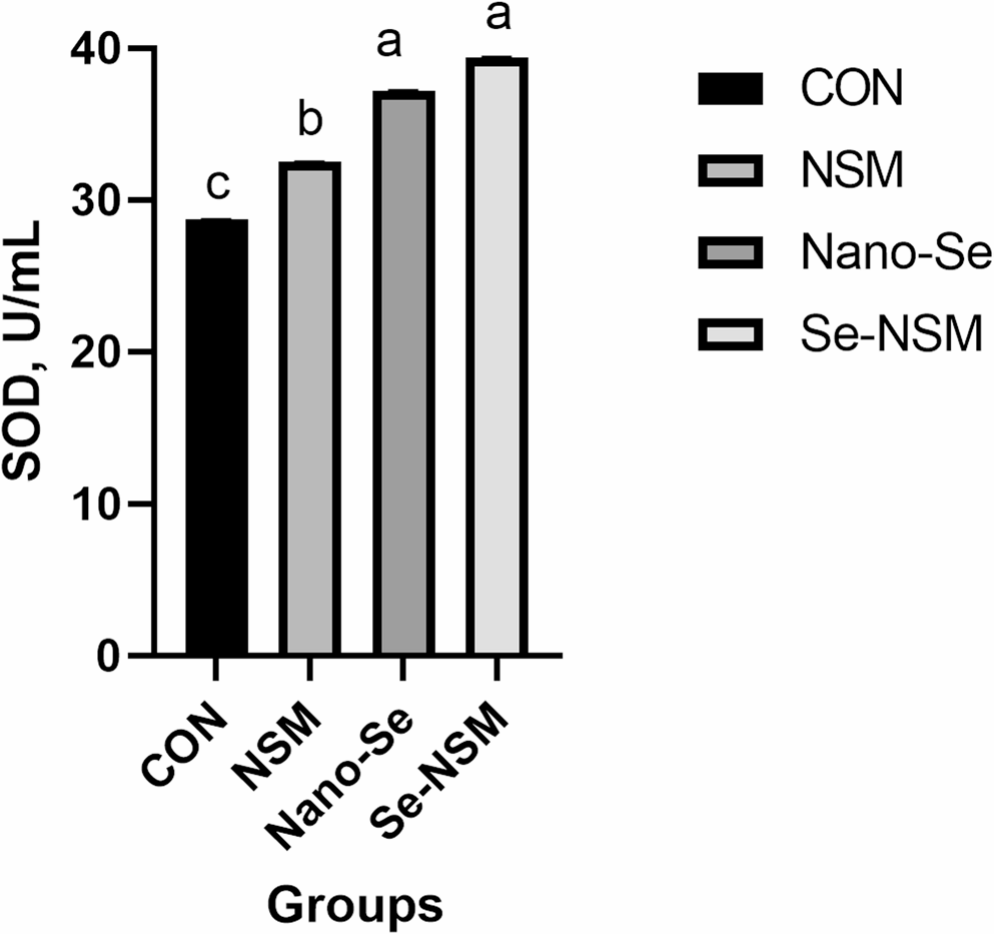
Effect of adding *Nigella sativa* meal and Nano-selenium on superoxide dismutase (SOD) levels in growing rabbits under hot environmental conditions. Growing rabbits were given a basal diet (CON, without feed additive), or supplemented with *Nigella sativa* meal (NSM, 40 g/kg), supplemented with Nano-selenium (Nano-Se, 0.1 mg/kg), supplemented with *Nigella sativa* meal and Nano-selenium (Se-NSM); Means with different superscripts within the same row differ significantly (a–d, *p* < 0.05); All data are expressed as the mean ± SD

**Fig. 4:**
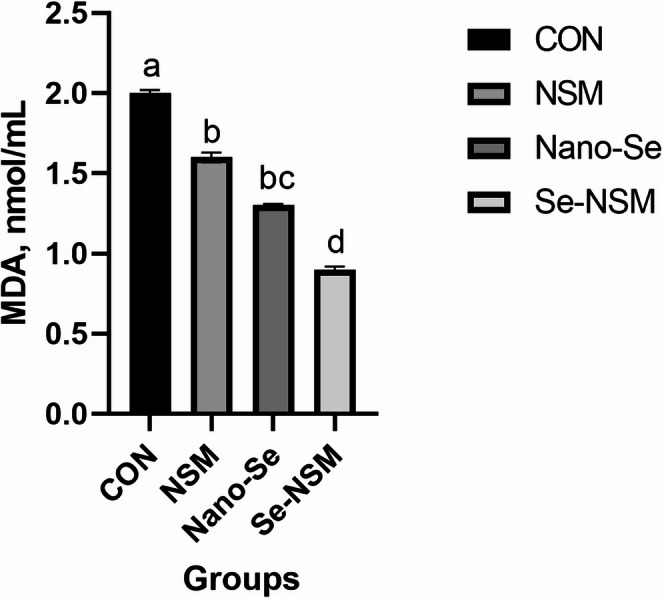
Effect of adding *Nigella sativa* meal and Nano-selenium on malondialdehyde (MDA) levels in growing rabbits under hot environmental conditions. Growing rabbits were given a basal diet (CON, without feed additive), or supplemented with *Nigella sativa* meal (NSM, 40 g/kg), supplemented with Nano-selenium (Nano-Se, 0.1 mg/kg), supplemented with *Nigella sativa* meal and Nano-selenium (Se-NSM); Means with different superscripts within the same row differ significantly (a–d, *p* < 0.05); All data are expressed as the mean ± SD

Blood biochemical constituents, including lipid profile, immunoglobulin level, hepatic enzyme activity, and antioxidative activity, are shown in Tables 5 and 6. The dietary addition of NSM, Nano-Se, and Se-NSM significantly increased glucose and total protein concentration compared to the control group. Feeding diets supplemented with NSM, Nano-Se, and Se-NSM significantly reduced AST, triglycerides, cholesterol, and LDL levels compared to the control group. No significant differences in ALT, HDL, and albumin levels were observed between the rabbits fed the treated diet and rabbits fed the control diet. Dietary supplementation with NSM, Nano-Se, and Se-NSM caused a significant increase in the IgA and IgG levels, while the IgM level was not affected significantly (*P* < 0.05) by the supplemental feeding of treated compared to the rabbits fed the control diet. Additionally, thyroid function improved, triiodothyronine (T3) levels increasing (*P* < 0.05) in rabbits that received NSM, Nano-Se, and Se-NSM compared to rabbits that received the control diet (Fig. 1). Also, the experimental supplements supported the oxidant status as shown in Figs. 2 and 3, and 4, as the serum SOD and TAC levels increased, meanwhile, the MDA level decreased (*P* < 0.05) in rabbits receiving NSM, Nano-Se, and Se-NSM compared to rabbits receiving the control diet.

**Table 5:** Effect of *Nigella sativa* meal and Nano-selenium-supplementation on blood profile in growing rabbits under hot environmental conditions

Parameters	CON	NSM	Nano-Se	Se-NSM	*P* value
Glucose (mg/dL)	61.2 ± 0.42^c^	66.4 ± 0.61^b^	67.8 ± 0.74^b^	69.3 ± 0.52^a^	0.001
Total protein (g/dL)	3.05 ± 0.15^c^	3.36 ± 0.19^ab^	3.23 ± 0.08^b^	3.49 ± 0.17^a^	0.004
Albumin (g/dL)	1.31 ± 0.04	1.32 ± 0.02	1.29 ± 0.05	1.34 ± 0.03	0.238
Triglycerides (mg/dL)	68.4 ± 0.35^a^	61.6 ± 0.67^b^	62.7 ± 0.51^b^	61.2 ± 0.48^b^	0.001
Cholesterol (mg/dL)	83.6 ± 0.24^a^	78.1 ± 0.31^b^	75.3 ± 0.82^bc^	71.6 ± 0.64^c^	< 0.001
LDL (mg/dL)	28.3 ± 0.06^a^	24.6 ± 0.04^c^	26.4 ± 0.05^b^	23.8 ± 0.03^c^	< 0.001
HDL (mg/dL)	35.4 ± 0.19	36.3 ± 0.25	35.5 ± 0.31	36.7 ± 0.14	0.104

Growing rabbits were given a basal diet (CON, without feed additive), or supplemented with *Nigella sativa* meal (NSM, 40 g/kg), supplemented with Nano-selenium (Nano-Se, 0.1 mg/kg), supplemented with *Nigella sativa* meal and Nano-selenium (Se-NSM); Means with different superscripts within the same row differ significantly (a–c, *p* < 0.05); All data are expressed as the mean ± SD

**Table 6:** Effect of *Nigella sativa* meal and Nano-selenium-supplementation on liver functions and immunity in growing rabbits under hot environmental conditions

Parameters	CON	NSM	Nano-Se	Se-NSM	*P* value
Liver functions					
AST (IU/l)	48.6 ± 1.34^a^	40.7 ± 0.97^c^	46.3 ± 1.06^b^	39.4 ± 1.56^c^	< 0.001
ALT (IU/l)	56.4 ± 2.18	54.2 ± 3.08	55.6 ± 2.25	53.8 ± 4.12	0.072
Immunity					
IgA (lg/mL)	21.3 ± 0.24^c^	25.1 ± 0.19^b^	24.5 ± 0.31^b^	27.2 ± 0.25^a^	< 0.001
IgM (lg/mL)	62.7 ± 0.52	63.4 ± 0.40	62.9 ± 0.63	63.7 ± 0.58	0.183
IgG (mg/mL)	2.43 ± 0.37^c^	3.15 ± 0.22^ab^	2.92 ± 0.41^b^	3.38 ± 0.29^a^	0.001

Growing rabbits were given a basal diet (CON, without feed additive), or supplemented with *Nigella sativa* meal (NSM, 40 g/kg), supplemented with Nano-selenium (Nano-Se, 0.1 mg/kg), supplemented with *Nigella sativa* meal and Nano-selenium (Se-NSM); Means with different superscripts within the same row differ significantly (a–c, *p* < 0.05); All data are expressed as the mean ± SD

### Microbial content

Table 7 illustrates the effect of adding *Nigella sativa* meal, Nano-selenium, or their mixture on intestinal microbial count in growing rabbits under hot environmental conditions. *Lactobacillus* counts increased in rabbits receiving supplements NSM, Nano-Se, and Se-NSM compared to the control diet. *Escherichia coli* counts decreased (*P* < 0.05) in rabbits receiving supplements NSM and Se-NSM compared to those receiving supplements in the control diet. *Clostridium perfringens* counts decreased (*P* < 0.05) in rabbits receiving supplements Se-NSM compared to the other groups.

**Table 7:** Effect of *Nigella sativa* meal and nano-selenium-supplementation on microbial enumeration in growing rabbits under hot environmental conditions

Parameters	CON	NSM	Nano-Se	Se-NSM	*P* value
*Lactobacillus*	4.6 ± 1.24^c^	6.7 ± 1.09^a^	5.8 ± 1.26^b^	6.9 ± 1.36^a^	0.001
*E. coli*	3.4 ± 2.18^a^	2.5 ± 3.08^b^	2.9 ± 2.25^ab^	1.8 ± 4.12^c^	< 0.001
*C. perfringens*	2.43 ± 0.37^a^	2.15 ± 0.22^ab^	2.52 ± 0.4^a^	1.78 ± 0.29^b^	0.001

Growing rabbits were given a basal diet (CON, without feed additive), or supplemented with *Nigella sativa* meal (NSM, 40 g/kg), supplemented with Nano-selenium (Nano-Se, 0.1 mg/kg), supplemented with *Nigella sativa* meal and Nano-selenium (Se-NSM); Means with different superscripts within the same row differ significantly (a–c, *p* < 0.05); All data are expressed as the mean ± SD

### Gene expression

**Fig. 5:**
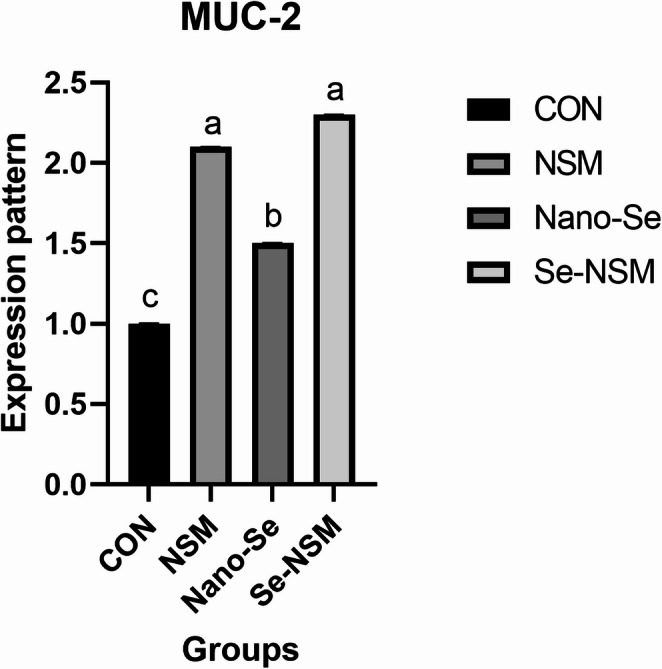
Effect of adding *Nigella sativa* meal and Nano-selenium on mucin-2 (MUC-2) levels in growing rabbits under hot environmental conditions. Growing rabbits were given a basal diet (CON, without feed additive), or supplemented with *Nigella sativa* meal (NSM, 40 g/kg), supplemented with Nano-selenium (Nano-Se, 0.1 mg/kg), supplemented with *Nigella sativa* meal and Nano-selenium (Se-NSM); Means with different superscripts within the same row differ significantly (a–d, *p* < 0.05); All data are expressed as the mean ± SD

**Fig. 6:**
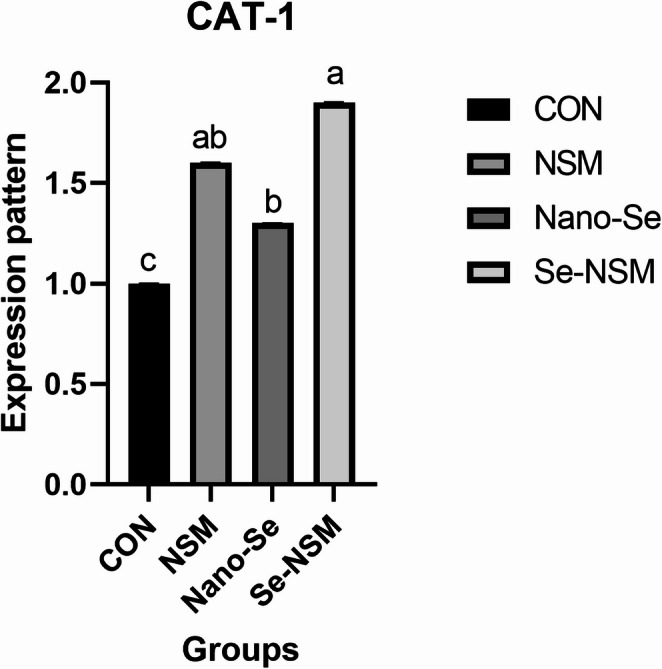
Effect of adding *Nigella sativa* meal and Nano-selenium on cationic amino acid transporter-1 (CAT-1) levels in growing rabbits under hot environmental conditions. Growing rabbits were given a basal diet (CON, without feed additive), or supplemented with *Nigella sativa* meal (NSM, 40 g/kg), supplemented with Nano-selenium (Nano-Se, 0.1 mg/kg), supplemented with *Nigella sativa* meal and Nano-selenium (Se-NSM); Means with different superscripts within the same row differ significantly (a–d, *p* < 0.05); All data are expressed as the mean ± SD

Figures 5 and 6 illustrate the effects of adding *Nigella sativa* meal and nano-selenium on the expression of MUC-2 and CAT-1 genes in rabbits exposed to hot environmental conditions. MUC-2 and CAT-1 gene expression was up-regulated in rabbits receiving supplements NSM, Nano-Se, and Se-NSM (*P* < 0.05) compared to the control group. The highest MUC-2 gene expression was in the group that received NSM and Se-NSM (*P* < 0.05), while the highest CAT-1 gene expression was in the group that received Se-NSM.

## Discussion

Heat stress causes considerable losses in the livestock sector by damaging intestinal tissues, impairing antioxidant and immune functions, reducing nutrient utilization efficiency, and increasing mortality rates, ultimately leading to poor performance. Therefore, the search for safe and effective nutritional strategies to mitigate the adverse effects of heat stress remains a critical area of research. Therefore, the current study was designed to investigate the potential role of *Nigella sativa* meal, Nano-selenium, and their mixture supplements as antioxidants, immunomodulatory, antimicrobials, and growth promoters, which may be a potential and effective anti-stress agent.

Results of the current study showed a significant improvement in the performance of heat-stressed rabbits fed diets supplemented with Se-NSM. In agreement with our results, Kassim et al. (2022) and Elbaz et al. (2025f) found a significant improvement in rabbits fed *Nigella sativa* meal or nano-selenium compared to the control group. Similarly, several reports have demonstrated the role of essential oils or nano-selenium in enhancing FCR and BWG in chickens exposed to heat stress (Abdel-Moneim et al. 2022; Elbaz et al. 2025a). The improved growth performance may be due to the essential oils’ content of active compounds that act as antioxidants and immunomodulatory and promote gut health by reducing intestinal inflammation, modifying microbial content, and upregulating certain intestinal genes (Elbaz et al. 2022; Ge et al. 2023). This improves the regenerative capacity of epithelial cells and increases the strength of the intestinal barrier against oxidative stress (Zeng et al. 2015), thus enhancing nutrient absorption and growth. A previous study also showed a decrease in rectal temperature in male rabbits treated with Se, in addition to improved serum glucose concentrations (El-Desoky et al. 2017), which contribute to improved physiological conditions by providing the energy needed for cellular metabolism without producing additional heat, indicating positive effects on metabolism during heat stress. Several reports have also demonstrated the role of Se in improving the scavenging activity of reactive oxygen species (Bernabucci et al. 2010; Hosny et al. 2020). Se is involved in forming functional selenogenic proteins (Hosny et al. 2020), including thioredoxin reductase, GSH-Px, and iodothyronine deiodinase, which reduces oxidative stress resulting from exposure to heat stress and thus enhances growth performance. Many studies have indicated that Se supplements improved growth performance in growing rabbits (El-Shobokshy et al. 2022), as its antioxidant properties prevented the harmful effects of free radicals and reduced pathogens. Therefore, Se-NSM supplements can serve as anti-heat stress agents due to their effectiveness in supporting immune response and enhancing gut health and antioxidant status, thus improving digestion, nutrient absorption, and growth performance of growing rabbits.

Interestingly, the experimental treatments did not affect the carcass characteristics of the rabbits (except for carcass weight) despite improved growth performance in rabbits fed Se-NSM. In the current study, carcass weight increased in rabbits fed a diet containing Se-NSM compared to the control group. These results are consistent with those of Attia et al. (2015), Ayyat et al. (2018), and Kassim et al. (2022), who found improved carcass weight in rabbits receiving essential oil or nano-selenium. Similarly, Elbaz et al. (2025f) found that feeding rabbits Nigella sativa meal led to an increase in carcass weight. The increased carcass weight in rabbits fed Se-NSM may be attributed to the role of the bioactive compounds in the essential oils in increasing the activity of digestive enzymes (Elbaz et al. 2025c) and improving intestinal integrity and function (Hosny et al. 2020), which increases nutrient availability, which is reflected in carcass characteristics. Additionally, selenium is essential for regulating oxidative stress, immunity, energy, and protein digestion, supporting nutrient digestion, absorption, and utilization (Zhang et al. 2011), and promoting normal animal growth. Therefore, adding *Nigella sativa* meal-nano selenium mixture (Se-NSM) can have a synergistic effect to support carcass characteristics in growing rabbits during heat stress.

The findings of the current study indicated that supplementation of Se-NSM in the diet of heat-stressed rabbits enhanced the digestibility of dry matter, crude fiber, and crude protein, and increased the activities of lipase and trypsin enzymes. These results were consistent with the findings of El-Shobokshy et al. (2022) and Dawood et al. (2020), who reported that adding selenium enhanced the digestion of nutrients. Additionally, many previous reports have indicated the positive role of aromatic plant supplements and their extracts in enhancing the digestion of dietary nutrients and increasing the activity of digestive enzymes (Elbaz et al. 2025b; Fathi et al. 2023). The improved nutrient digestibility may be attributed to the numerous properties of both Nano-selenium and *Nigella sativa*, including antimicrobial and gut health-promoting properties (Ibrahim et al. 2020; Fathi et al. 2023), as well as their role in enhancing digestive enzyme activity (Elbaz et al. 2022), which enhances the metabolism of nutrients. It was also shown that adding medicinal and aromatic plants and their products to the diet significantly improved the length of the small intestine (Chen et al. 2024a, b), as well as the tendency to enhance the intestinal morphology through the increase of intestinal villi, which enhances intestinal function (Choi et al. 2020; Wang et al. 2022) and absorption capacity, thus increasing nutrient availability, which increases carcass weight. These results demonstrate the effective role of adding a mixture of *Nigella sativa* meal and Nano-selenium in enhancing feed efficiency for rabbits exposed to heat stress, which maintains animal health and increases growth rate.

Heat stress causes severe damage to fat metabolism, oxidation, and hepatocyte enzyme secretion (ALT and AST), leading to deterioration in animal health. Therefore, it was necessary in the current study to investigate the effect of heat stress and experimental supplements on fat metabolism and liver health. Our results showed a significant improvement in the physiological performance of rabbits fed *Nigella sativa* meal-nano selenium mixture (Se-NSM), as glucose and total protein levels increased, while triglycerides, cholesterol, LDL, and AST levels decreased compared to rabbits fed the control diet. Several reports support our findings, as Qin et al. (2016) and Kassim et al. (2022) found significant improvements in liver and kidney functions, significantly improved ALT, AST, total protein, glucose, triglycerides, and cholesterol levels in poultry and rabbits receiving selenium. These results are consistent with those of Hassan et al. (2019) and Fathi et al. (2023), who reported that *Nigella sativa* significantly reduced total triglyceride and cholesterol levels. Likewise, Elbaz et al. (2025f) found a similar effect of black seed meal supplements, which led to a reduction in cholesterol, LDL, and triglyceride levels and an increase in HDL levels in the blood of rabbits. The improved blood lipid profile of rabbits receiving Se-NSM may be due to *Nigella sativa’s* high content of unsaturated fatty acids and sterols, which may stimulate intestinal cholesterol secretion and inhibit dietary cholesterol absorption (Khan et al. 2012). Alternatively, *Nigella sativa’s* active compounds may indirectly influence a key enzyme (HMG-CoA reductase) involved in cholesterol synthesis (Elbaz et al. 2025d). Furthermore, several reports have revealed liver dysfunction under stress conditions, which is caused by the leakage of enzymes from the cytoplasm of degenerating and dying cells, leading to increased liver enzyme levels. In this study, liver enzyme concentrations decreased in the serum of rabbits receiving Se-NSM, consistent with the findings of Kassim et al. (2022) and Fathi et al. (2023). This may indicate that the mixture of *Nigella sativa* meal and nano-selenium has properties that promote liver health and serum lipid profiles in heat-stressed rabbits.

Maintaining the health of the thyroid gland is essential due to its crucial role in many vital processes, such as thermoregulation, digestion, metabolism, and energy maintenance, as well as its effective role in immune system function (Lin et al. 2008). Thyroid hormone production is influenced by many factors, including ambient temperature and nutritional composition (Abdel-Moneim et al. 2021; Bayraktar et al. 2023). The results of our study showed a significant effect on thyroid activity, as T3 production was significantly increased with the addition of the mixture of *Nigella sativa* meal and nano-selenium. These results are similar to those of Bayraktar et al. (2023) and Elbaz et al. (2025d, f), who reported that the addition of essential oils stimulated the production of thyroid hormones (T3 and T4), which may be due to the physiological role of the oil in increasing the modification of the intestinal microbiota, which promotes the secretion of corticotropin, which plays a key role in stimulating the production of thyroid hormones (thyrotropin). In contrast, Wang et al. (2025) found no effect of essential oil supplements on thyroid hormone production. This discrepancy may be due to the type and concentration of essential oil, the age of the chickens, and their health status. Therefore, experimental supplements may have an effective impact in enhancing growth performance by increasing T3 secretion, which positively impacts digestion, metabolism, and thermoregulation by increasing the absorptive capacity of the intestinal mucosa as a result of morphological changes or increased activity of intestinal enzymes (Yilmaz and Gul 2024), thereby increasing available nutrients.

Heat stress leads to many harmful changes, including weakening of the immune system, which is represented by a decrease in the relative weight of immune and reproductive organs, as well as a decline in antibody levels (Abdel-Moneim et al. 2021; Madkour et al. 2023). Immunoglobulins (IgM, IgG, and IgA) play a crucial role in the immune system; they are important antibodies in the immune response, especially when growing rabbits are exposed to stress. In the present study, IgG and IgA levels in the Se-NSM group were significantly increased, indicating the potential protective effects of the *Nigella sativa* meal and nano-selenium combination on growing rabbits during heat stress. Consistent with these findings, several reports have found an immune-enhancing effect of medicinal plant supplements and their products through increased levels of immunoglobulins (Wang et al. 2024; Elbaz et al. 2025a, d). Similarly, Kilany et al. (2023) found that adding nano-selenium increased levels of immunoglobulins.

During heat stress, an imbalance between oxidation and antioxidant activity occurs in the animal’s body, leading to the production of various reactive oxygen species (ROS), which causes exposure to oxidative stress, which causes tissue damage, and disease progression. Heat stress depletes antioxidant reserves and reduces the activity of mitochondrial enzymes, as well as their oxidative capacity (Madkour et al. 2023; Yilmaz and Gul 2024). The first line of defense against ROS during stress is antioxidant enzymes (including SOD, GSH-Px, and CAT), which are commonly used as biomarkers of oxidative stress and the role of experimental supplements in mitigating oxidative stress. Although the bioactive components of *Nigella sativa* meal were not analyzed in the present study, their composition and biological functions have been well-documented in previous study (Ge et al. 2023; Yilmaz and Gul 2024), which supports their potential contribution to the observed antioxidative and immunomodulatory effects. Our study found an increase in the activity of antioxidant enzymes such as SOD and TAC in growing rabbits receiving Se-NSM. Consistent with our study, several previous studies have found that *Nigella sativa* meal or nano-selenium supplements significantly enhanced the antioxidant capacity of growing rabbits by increasing levels of SOD, CAT, and GPx. The improvement in antioxidant capacity with the addition of the mixture may be attributed to the bioactive compounds in the essential oils, in addition to the antioxidant properties of nano-selenium (Kilany et al. 2023; Elbaz et al. 2022). This is especially true since selenium is the active component of GPX, which plays a crucial role as an antioxidant (Zhang et al. 2011). Antioxidants, such as the active compounds in essential oils, also exhibit a high reactivity with peroxyl radicals and are eliminated through the transfer of formal hydrogen atoms (Yilmaz and Gul 2024). However, feeding growing rabbits Se-NSM resulted in decreased serum MDA levels, which is consistent with several reports (Wang et al. 2018; Abdel-Moneim et al. 2022). MDA levels are used to determine the level of tissue damage resulting from oxidative stress, the major final product of lipid peroxidation (Attia et al. 2017; Elbaz 2023). The decrease in MDA levels in the current study when rabbits were fed a mixture of *Nigella sativa* meal and nano-selenium indicates the mixture’s role in maintaining cellular integrity. The above shows the enhancing effect of the mixture’s ability to mitigate the effects of oxidative stress in rabbits exposed to stress.

Heat stress causes excessive panting and superficial vasodilation, which leads to oxygen deficiency and organ perfusion deficiency, damaging the digestive system and its functions (Kim et al. 2021; Abdel-Moneim et al. 2021; Elnesr and Abdel-Azim 2023). Among the harmful effects on the digestive system are microbial imbalance, suppressed immunity, altered gene expression, and damage to intestinal tissue, as well as adverse effects on digestion and nutrient absorption (Elnesr and Abdel-Azim 2023; Elbaz et al. 2025b, c). The current study showed an imbalance in the intestinal microbial content (increased *E. coli* and *C. perfringens* counts) and decreased expression of MUC-2 and CAT-1 gene in rabbits exposed to hot conditions, which is consistent with several reports (Elbaz et al. 2025b; Abdel-Moneim et al. 2022). Nevertheless, rabbits that received the Se-NSM showed an increase in *Lactobacillus* counts, a decrease in *E. coli* and *C. perfringens* counts, and an increase in MUC-2 and CAT-1 gene expression. Similarly, in rabbit nutrition, essential oils and plant extracts have shown positive results in alleviating post-weaning challenges by modifying the microbial balance in the digestive tract, thus enhancing intestinal integrity (Elwan et al. 2019; Pugliese et al. 2024). Consistent with our findings, a previous study demonstrated that *Nigella sativa meal* altered the diversity of the gut microbiome by reducing pathogenic microbial load by inhibiting *E. coli* (Elbaz et al. 2025f). Moreover, feeding selenium-fortified feed significantly modified the intestinal microbiota, with a significant reduction in the number of pathogenic bacteria (Al-Sagheer et al. 2023). Furthermore, several reports indicate that the bioactive compounds in essential oils play a crucial role in improving the efficiency of nutrient absorption in the gastrointestinal tract of rabbits by modulating microbial balance, microbial fermentation, and intestinal motility (Elwan et al. 2019; Pugliese et al. 2024). Additionally, in this study, Nano-Se and *Nigella sativa* meal supplementation increased mRNA expression of MUC-2, consistent with the findings of Chen et al. 2024a, b; Elbaz et al. (2025f). Previous studies have also shown that the addition of medicinal and aromatic plants and their products had modulatory effects on certain genes, such as MUC-2 and CAT-1 (Ruan et al. 2021; Elbaz et al. 2025b, c, f). Likewise, Pu et al. (2018) found increased expression of the MUC-2 gene in pigs fed a diet containing oregano oil. Goblet cells synthesize MUC-2, demonstrating the role of selenium and *Nigella sativa* meal in significantly enhancing goblet cell density and differentiation and improving mucus structure in animals (Elbaz et al. 2025f). Elevated CAT-1 gene expression reflects enhanced absorption of cationic amino acids from the feed (Liu et al. 2019). This positive effect on the gut microbiome and genetic modification supports heat-stressed rabbit health by inhibiting pathogen colonization and restoring the integrity and function of the intestinal barrier, thereby enhancing nutrient digestion and absorption (Pu et al. 2018; Aliakbarpour et al. 2012).

## Conclusions

The Se-NSM mixture enhanced intestinal health via regulating the gene expression of MUC2 and CAT-1 and modifying the microbial content in heat-stressed growing rabbits. Additionally, the mixture enhanced the immune response and antioxidant status of growing rabbits exposed to environmental heat stress. Moreover, the mixture increased nutrient digestibility, carcass characteristics, and body weight gain and enhanced the feed conversion ratio, which improved the productive performance of heat-stressed growing rabbits. Therefore, adding the mixture of *Nigella sativa* meal (40 g/kg) and Nano-selenium (0.1 mg/kg) to the diet of growing rabbits exposed to environmental heat stress had effective effects in enhancing health and performance, which may be an effective nutritional strategy for combating heat stress.

## Data Availability

All the data supporting this article are available from the corresponding author upon request.
